# WZ-3146 acts as a novel small molecule inhibitor of KIF4A to inhibit glioma progression by inducing apoptosis

**DOI:** 10.1186/s12935-024-03409-y

**Published:** 2024-06-27

**Authors:** Tao Yan, Qing Jiang, Guangpu Ni, Haofeng Ma, Yun Meng, Guiqiong Kang, Meifang Xu, Fei Peng, Huadong Li, Xin Chen, Mingguang Wang

**Affiliations:** 1 Central Laboratory, Linyi People’s Hospital, Linyi, Shandong Province 276000 China; 2 Linyi Key Laboratory of Neurophysiology, Linyi People’s Hospital, Linyi, Shandong Province 276000 China; 3 Department of Neurosurgery, First Affiliated Hospital of Harbin Medical University, Harbin, Heilongjiang Province 150001 China; 4 Key Laboratory of Neurosurgery of Colleges and Universities in Heilongjiang Province, Harbin, Heilongjiang Province 150001 China; 5 Department of Neurosurgery, Linyi People’s Hospital, Shandong Second Medical University, Linyi, Shandong Province 276000 China; 6 Department of Neurology, Linyi People’s Hospital, Shandong Second Medical University, Linyi, Shandong Province 276000 China; 7 Department of Neurosurgery and Neurosurgical Disease Research Centre, the Second Affiliated Hospital of Guangzhou Medical University, Guangzhou, Guangdong Province China

**Keywords:** Glioma, Targeted therapy, KIF4A, WZ-3146

## Abstract

**Background:**

Glioma is considered the most common primary malignant tumor of the central nervous system. Although traditional treatments have not achieved satisfactory outcomes, recently, targeted therapies for glioma have shown promising efficacy. However, due to the single-target nature of targeted therapy, traditional targeted therapies are ineffective; thus, novel therapeutic targets are urgently needed.

**Methods:**

The gene expression data for glioma patients were derived from the GEO (GSE4290, GSE50161), TCGA and CGGA databases. Next, the upregulated genes obtained from the above databases were cross-analyzed, finally, 10 overlapping genes (BIRC5, FOXM1, EZH2, CDK1, KIF11, KIF4A, NDC80, PBK, RRM2, and TOP2A) were ultimately screened and only KIF4A expression has the strongest correlation with clinical characteristics in glioma patients. Futher, the TCGA and CGGA database were utilized to explore the correlation of KIF4A expression with glioma prognosis. Then, qRT-PCR and Western blot was used to detect the KIF4A mRNA and protein expression level in glioma cells, respectively. And WZ-3146, the small molecule inhibitor targeting KIF4A, were screened by Cmap analysis. Subsequently, the effect of KIF4A knockdown or WZ-3146 treatment on glioma was measured by the MTT, EdU, Colony formation assay and Transwell assay. Ultimately, GSEA enrichment analysis was performed to find that the apoptotic pathway could be regulated by KIF4A in glioma, in addition, the effect of WZ-3146 on glioma apoptosis was detected by flow cytometry and Western blot.

**Results:**

In the present study, we confirmed that KIF4A is abnormally overexpressed in glioma. In addition, KIF4A overexpression is a key indicator of glioma prognosis; moreover, suppressing KIF4A expression can inhibit glioma progression. We also discovered that WZ-3146, a small molecule inhibitor of KIF4A, can induce apoptosis in glioma cells and exhibit antiglioma effects.

**Conclusion:**

In conclusion, these observations demonstrated that targeting KIF4A can inhibit glioma progression. With further research, WZ-3146, a small molecule inhibitor of KIF4A, could be combined with other molecular targeted drugs to cooperatively inhibit glioma progression.

**Supplementary Information:**

The online version contains supplementary material available at 10.1186/s12935-024-03409-y.

## Introduction

Glioma is the most common primary malignant tumor of the central nervous system and has high mortality and disability rates [1–3]. Moreover, the five-year survival rate of glioma patients is unsatisfactory even after surgery combined with standard chemoradiotherapy [4]. Because of the high invasiveness of glioma, its complete resection is difficult [5]. In addition, because of the changes in multiple genes and pathways within glioma cells, favorable outcomes are difficult to achieve with traditional chemotherapeutic approaches. Therefore, new methods for glioma treatment are urgently needed.

The alteration of multiple genes and pathways is an important characteristic of glioma that not only affects the efficacy of traditional chemotherapeutic drugs but also is closely related to glioma progression [6]. Several genetic alterations, including alterations in the genes encoding vascular endothelial growth factor (VEGF), platelet-derived growth factor (PDGF) and their receptors (VEGFR and PDGFR), have been confirmed in glioma, these genetic alterations are often considered to be involved in the malignant progression of glioma cells by affecting processes such as proliferation, growth, apoptosis and invasion. For example, VEGFR1 and VEGFR2 are believed to be critical factors in glioblastoma progression, and VEGF overexpression can promote abnormal vascular proliferation in glioblastoma multiforme (GBM) by binding to its receptors VEGFR1 and VEGFR2 [7, 8]. In glioblastoma, the Ras pathway has been demonstrated to act as an important signal transduction effector of PDGFR, and abnormal mutation or amplification of PDGFR can trigger MAPK signaling by overactivating Ras, ultimately leading to cytoskeletal remodeling, cell proliferation and proangiogenic growth factor release [9]. In summary, these evidences confirm that the abnormal expression of oncogenes is a crucial factor in glioma progression, and attempts to target these abnormal changes in oncogenes and related pathways is anticipated to become a feasible treatment strategy for glioma.

Targeted therapy, which can directly target oncogenes and related pathways to increase sensitivity to tumor therapy, reduce antitumor drug resistance, and decrease cytotoxicity, is one of the hotspots in tumor therapy research. For example, EGFRvIII is the most common mutation of EGFR and can potentially be used as a marker for glioblastoma treatment [10]. Erlotinib, a selective EGFR inhibitor, can significantly inhibit the proliferation of glioma cells with the EGFRvIII mutation by reducing EGFRvIII kinase activity [11], and it has been observed that erlotinib has a good therapeutic effect on adult high-grade gliomas [12]. BRAF has carcinogenic effects on various organs and tissues [13–15]. The BRAFV600E mutation is the most common BRAF mutation in primary brain tumors and has been found in adult and pediatric gliomas, Dabrafenib, an effective BRAFV600 inhibitor, has shown therapeutic effects on BRAFV600-mutated pediatric gliomas [16]. Selumetinib, a MEK inhibitor, can prolong the survival of patients with low-grade glioma [17–19]. Therefore, research on glioma-targeted therapy is anticipated to have great potential for clinical translation.

However, due to variability and heterogeneity of gliomas, single-agent targeted therapies have not shown significant therapeutic effects on glioma, and the results of clinical trials of first-generation targeted drugs have been disappointing. Consequently, the search for new glioma targets and the selection of the most likely small molecule inhibitors based on the new targets is anticipated to greatly benefit glioma treatment.

## Materials and methods

### Cell culture

U251, LN229, SF295, and TJ905 cell lines and HUVEC cell line were obtained from the Central Laboratory of Linyi People’s Hospital. The glioma cell lines were cultured in Dulbecco’s modified Eagle’s medium (DMEM; C111995500BT, Gibco, USA) supplemented with 1% dual antibiotic (Penicillin‒Streptomycin Solution, P1400, Solarbio, China) and 10% fetal bovine serum (FBS; 10270-106, Gibco, USA). HUVECs were cultured in Roswell Park Memorial Institute 1640 (RPMI-1640) medium (R8758, Sigma, USA) supplemented with 10% FBS and 1% dual antibiotic solution. All cell lines were cultured at 37 °C and 5% CO2 in a cell culture incubator.

### MTT assay

U251 and LN229 glioma cells were transfected with KIF4A siRNA and treated with WZ-3146. After 48 h, 10 µl of MTT solution (5 mg/ml) was added to the tissue culture plate (96-well), and the plate was cultured for 4 h in a 37 °C incubator. Next, the original culture medium was replaced with 150 µl of DMSO. Finally, cell viability was evaluated by measuring the absorbance at 490 nm using a SpectraMax M5 microplate reader.

### EdU assay

U251 and LN229 glioma cells were transfected with siKIF4A or treated with WZ-3146. The effects of siKIF4A and WZ-3146 on glioma cell proliferation and viability were measured with an EdU assay kit (Beyotime, China) according to the instructions.

### Colony formation assay

Suspensions of U251 and LN229 glioma cells were prepared. Afterward, the cells were seeded into a 6-well cell culture dish at 1000 cells per well. The cells were subsequently transfected with siKIF4A or treated with WZ-3146. After 10 days, the cells were fixed with 4% paraformaldehyde for 15 min and stained with crystal violet.

### Western blotting

The spare cells were placed on ice and lysed using RIPA buffer for 30 min. Total protein was extracted from the cell lysate. The proteins in the samples were separated on 12.5% or 7.5% SDS‒PAGE gels, and the membranes were then incubated with primary and secondary antibodies. Ultimately, the immunoreactions were detected with a FluorChem E imaging system. The primary antibodies used were as follows: anti-KIF4A (A10193, ABclonal), anti-Bcl-2 (68103-1-Ig, Proteintech), anti-BAX (50599-2-Ig, Proteintech), and anti-β-actin (TA-09, ZSGB-BIO).

### Cell transfection

According to the manufacturer’s instructions, Lipofectamine 8000 reagent (C0533, Beyotime, China) and siRNA were mixed at a defined ratio to obtain the working solution. Then, the glioma cell lines were transfected with the working solution. The KIF4A siRNAs were obtained from General Biosystems (China). The sequences of siKIF4A were shown in Table 1.

**Table 1:** Sequence of siRNA, primer

siRNA sequence	
siNC	5′ UUCUCCGAACGUGUCACGUTT 3′.
KIF4A-siRNA1	5′ GGAUGAAGAACUUGAGAAATT 3′.
KIF4A-siRNA2	5′ GCAUAAAGAUUGUGGGACUTT 3′.
KIF4A-siRNA3	5′ GCAAGAAGCCCAAGUAGAATT 3′.
Primer sequence	
GAPDH	F-5′ GCACCGTCAAGGCTGAGAAC 3′, R-5′ TGGTGAAGACGCCAGTGGA3′
KIF4A	F-5’TACTGCGGTGGAGCAAGAAG 3′, R-5’CATCTGCGCTTGACGGAGAG3′

### Plasmid transfection

According to the reagent instructions, the appropriate amount of plasmid is mixed with Lipofectamine 8000 reagent to form a complex. Then, the complex was used to infect glioma cells. After transfection, the fresh culture medium was used to replace the complex. The KIF4A overexpressed plasmid were derived from General Biosystems (China).

### qRT‒PCR

TRIzol reagent (T9424, Sigma, USA) was used to isolate total RNA. For reverse transcription, Evo M-MLV RT Master Mix (AG11706, Accurate Biology, China) was used. Afterward, the cDNA was used as a template for gene amplification using SYBR Green Pro Taq HS Premix (AG11701, Accurate Biology, China), and an ABI Prism 7500 rapid thermal cycling instrument (Applied Biosystems, CA, USA) was used to acquire gene amplification data. The sequences of the primers targeting KIF4A and GAPDH were shown in Table 1.

### Transwell assays

A total of 2 × 10^4 glioma cells were suspended in 200 µl of FBS-free DMEM and then seeded on Transwell filter membranes without (migration assay) or with (invasion assay) a Matrigel coating in the upper chamber (8.0 μm pore, JET, China). The lower chamber contained 400 µl of culture medium supplemented with 10% FBS. After incubation for 48 h, the cells on the lower surface of the membrane in each upper chamber were fixed with 4% paraformaldehyde for 30 min and then stained with 0.1% crystal violet for 15 min. The cells that migrated or invaded to the lower side of the membrane in each upper chamber were visualized by imaging with a Nikon Eclipse Ti microscope.

### Flow cytometry

To assess the effect of WZ-3146 on glioma cell apoptosis, cells were stained using an Annexin V-FITC cell apoptosis detection kit (Beyotime, China) according to the manufacturer’s instructions. The data were acquired and analyzed with a BD FACSCanto.

### Bioinformatics analysis and statistical analysis

Gene expression data for glioma patients were obtained from the GEO (GSE4290, GSE50161), TCGA and CGGA databases. The survival information for the glioma patients, including the glioma grade, IDH mutation status, 1p19q codeletion status and patient age, was obtained from the CGGA database. Prism version 7.0 was used to analyze intergroup differences. R packages obtained via http://bioconductor.org/ were used for bioinformatics analysis with R version 4.2.0. In the figures, different numbers of asterisks represent *P* < 0.001,*P* < 0.01, and *P* < 0.05.

## Results

### KIF4A was highly expressed in glioma

**Fig. 1:**
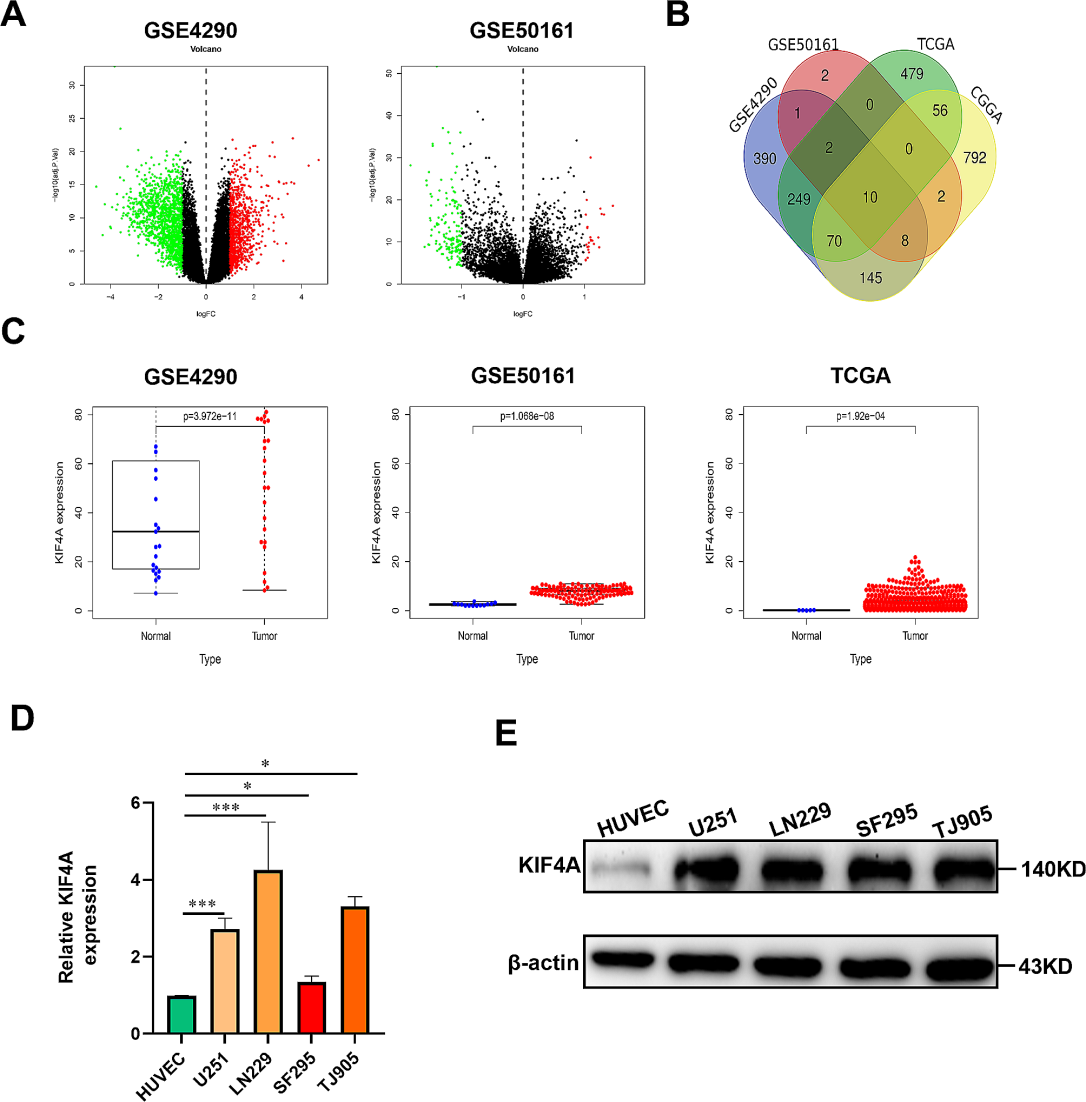
KIF4A was highly expressed in glioma. (**A**) Volcano plot of the genes expression in glioma from the GEO (GSE4290, GSE50161). (**B**) Venn diagram indicating the up-regulation of genes related to glioma progression from the GEO (GSE4290, GSE50161), TCGA and CGGA databases. (**C**) The expression of KIF4A in glioma samples from the GEO (GSE4290, GSE50161) and TCGA databases. (**D**) The relative KIF4A expression levels in HUVECs and U251, LN229, SF295 and TJ905 cells were measured by qRT‒PCR. (**E**) The relative KIF4A protein levels in HUVECs and U251, LN229, SF295 and TJ905 cells were measured by Western blotting. The bars indicate the means ± SDs; *P < 0.05, **P < 0.01, and ***P < 0.001

Tumor occurrence is closely related to the differential expression of multiple genes and differential activity of multiple pathways. To identify the crucial genes associated with glioma progression, gene expression data for glioma were obtained from the GEO (GSE4290, GSE50161) and TCGA databases (Fig. 1A). Next, the upregulated genes obtained from the above databases were cross-analyzed, and 12 overlapping upregulated genes (BIRC5, FOXM1, EZH2, CDK1, KIF11, KIF4A, NDC80, PBK, CPXM1, RRM2, TOP2A, and UHRF1) were identified. To further identify the genes associated with glioma progression, the corresponding mRNA expression data and survival-related information were first obtained from the CGGA database. After a series of analyses, including survival analysis, independent prognostic analysis, receiver operating characteristic (ROC) curve analysis, and clinical characteristic correlation analysis, the intersection of these identified genes with the abovementioned 12 genes was identified via Venn diagrams, and 10 overlapping genes (BIRC5, FOXM1, EZH2, CDK1, KIF11, KIF4A, NDC80, PBK, RRM2, and TOP2A) were ultimately identified. Finally, KIF4A expression was found to have the strongest correlations with the clinical characteristics of glioma patients, and KIF4A was eventually selected as the object for subsequent research (Fig. 1B). Subsequently, the GEO (GSE4290, GSE50161) and TCGA databases were used to analyze the expression of KIF4A in glioma, and the results showed that KIF4A was significantly overexpressed in glioma (Fig. 1C). Next, the qRT‒PCR and western blot analysis results indicated that the expression of KIF4A in glioma cell lines (U251, LN229, SF295, and TJ905) was greater than that in HUVECs (Fig. 1D‒E). Taken together, these results suggest that KIF4A is overexpressed in glioma and may be associated with glioma progression.

### KIF4A over-expression was associated with glioma prognosis and could be an independent prognostic factor for glioma

**Fig. 2:**
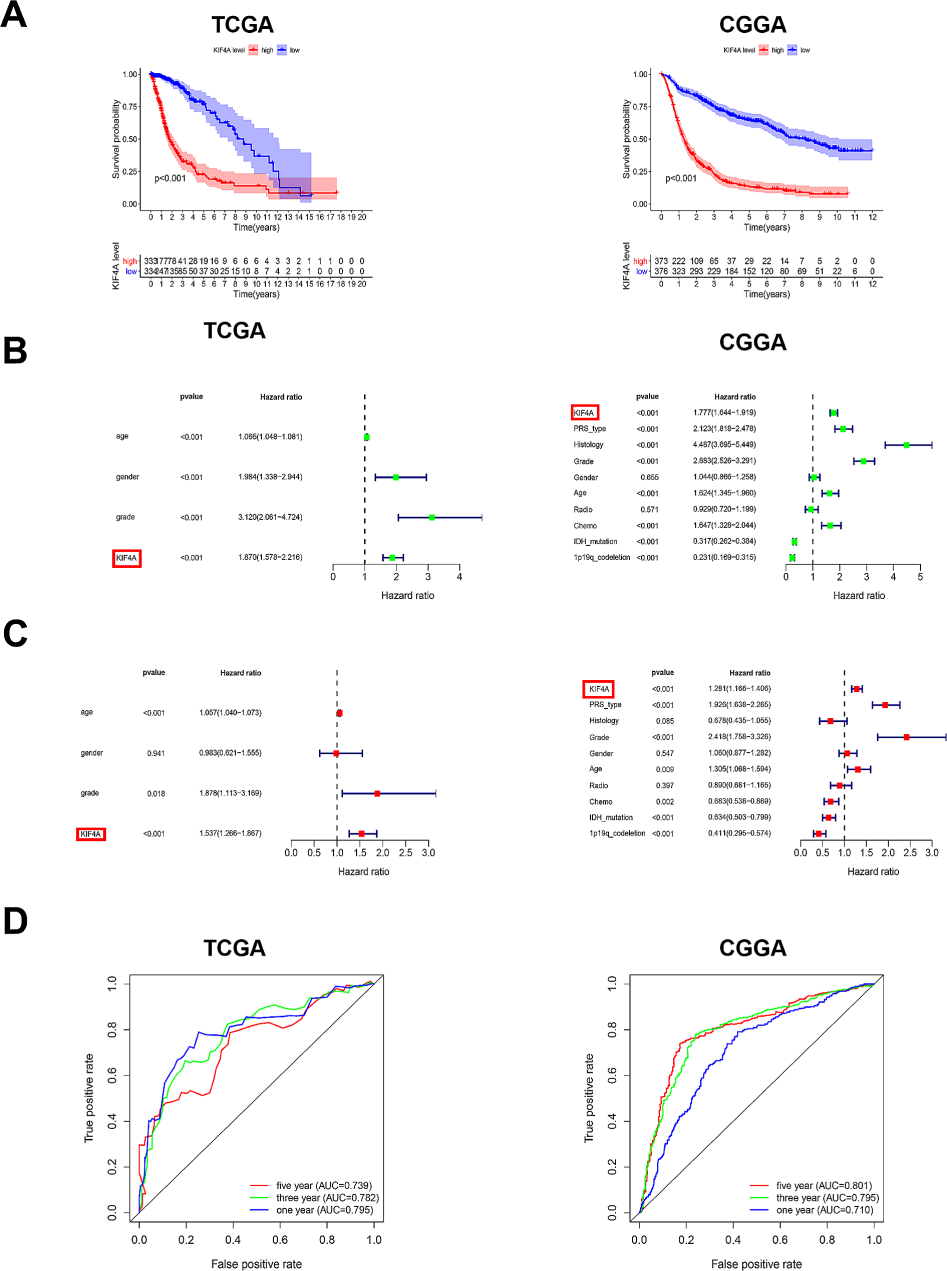
KIF4A overexpression was associated with poor prognosis in glioma patients. (**A**) Kaplan‒Meier survival analysis in glioma patients represented in the TCGA and CGGA databases. (**B-C**) Univariate and multivariate Cox regression analysis of KIF4A expression, with the hazard ratio (HR) among glioma patients in the TCGA and CGGA databases.(**D**) ROC curves for predicting one-year, three-year and five-year survival outcomes based on KIF4A expression in glioma patients in the TCGA and CGGA databases. The bars indicate the means ± SDs; *P < 0.05, **P < 0.01, and ***P < 0.001

To further investigate the correlation between KIF4A expression and glioma prognosis, KIF4A expression and survival information for patients with glioma were obtained from the TCGA and CGGA databases. Kaplan‒Meier survival analysis demonstrated that glioma patients with high KIF4A expression had shorter survival times than patients with low KIF4A expression. Next, univariate and multivariate Cox regression analysis were carried out to confirm the feasibility of KIF4A as an independent prognostic factor for glioma (Fig. 2A). Univariate Cox regression analysis indicated that KIF4A expression was negatively related to glioma prognosis in the TCGA database (HR = 1.870, *P* < 0.001), and similar results were obtained in the CGGA database (HR = 1.777, *P* < 0.001) (Fig. 2B). These results preliminarily suggested that KIF4A upregulation may be an independent risk factor for glioma prognosis. The subsequent multivariate Cox regression analysis results showed that KIF4A overexpression was a risk indicator for glioblastoma in both the TCGA and CGGA databases (TCGA: HR = 1.537, *P* < 0.001; CGGA: HR = 1.281, *P* < 0.001) (Fig. 2C). Overall, these results supported the idea that high KIF4A expression is an independent factor indicating poor prognosis in glioma. Furthermore, ROC curves were generated to assess the clinical diagnostic value of KIF4A for glioma in the TCGA and CGGA databases. For ROC curves, the area under the curve (AUC) represents the accuracy of a given variable for predicting prognosis, and an AUC greater than or equal to 0.7 indicates that the variable can accurately predict prognosis. As shown in the Fig. 2D, the AUCs of the ROC curves for predicting the 1-year, 3-year, and 5-year survival outcomes of glioma patients in the TCGA and CGGA databases were all greater than or close to 0.7, suggesting that KIF4A expression can be used to predict glioma patient survival. In summary, these results indicate that high KIF4A expression is significantly associated with poor prognosis in glioma patients and can also be used as a predictor of glioma prognosis.

### KIF4A overexpression was correlated with multiple clinical characteristics in glioma patients

**Fig. 3:**
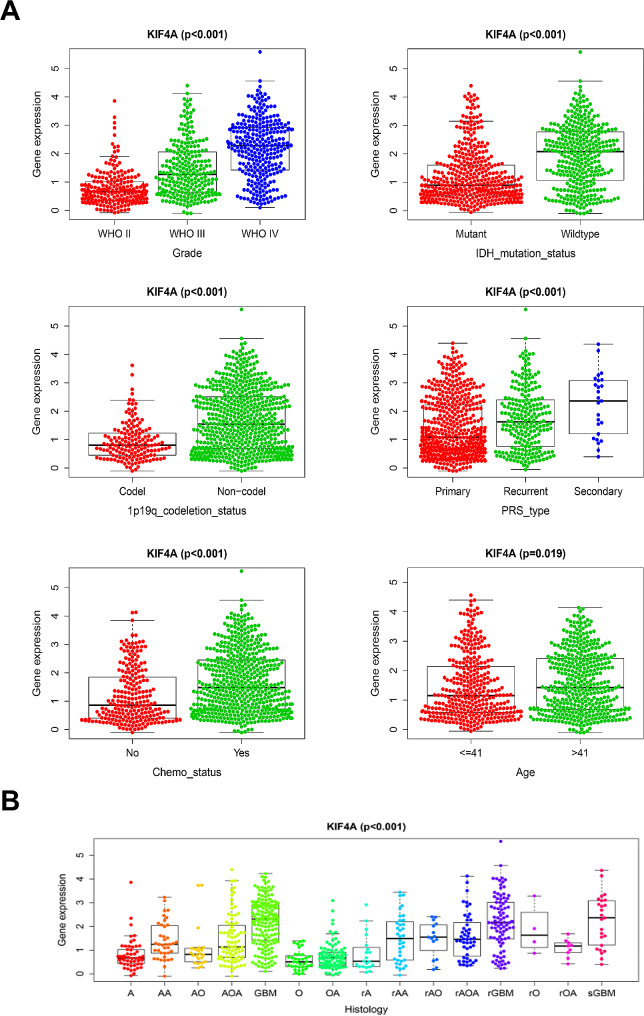
KIF4A overexpression was correlated with multiple clinical characteristics in glioma patients. (**A**) Relationships between KIF4A expression and relevant clinical information, including the glioma grade, IDH mutation status, 1p19q codeletion status, recurrent status, Chemotherapy status and patient age, according to the CGGA database. (**B**) Relationships between KIF4A expression and histological grade and prognosis in glioma patients in the CGGA database. The bars indicate the means ± SDs; *P < 0.05, **P < 0.01, and ***P < 0.001

Considering that KIF4A overexpression is associated with poor prognosis in glioma patients, the correlations between KIF4A expression and glioma clinical characteristics were further elucidated. Subsequently, KIF4A expression data, as well as relevant clinical information, including the glioma grade, IDH mutation status, 1p19q codeletion status and patient age, was extracted from the CGGA database. KIF4A expression was positively correlated with glioma grade. Reports have demonstrated that glioma patients with wild-type IDH have a worse prognosis than those with IDH mutation [20], and our results confirmed that KIF4A overexpression in patients with wild-type IDH was greater than that in patients with IDH mutation. This result was consistent with the finding that glioma patients with high KIF4A expression had a worse prognosis. Similarly, the 1p19q codeletion status is also correlated with glioma prognosis, and glioma patients with 1p19q codeletion often have a better prognosis [21]. Our analysis revealed that the expression of KIF4A was significantly decreased in glioma patients with 1p19q codeletion and that KIF4A was significantly upregulated in patients with recurrent glioma. And, KIF4A expression was associated with the Chemotherapy status. In addition, as the patient age increased, KIF4A expression increased significantly. Additionally, KIF4A overexpression was significantly associated with a high histological grade and poor prognosis in glioma patients (Fig. 3A-B). Taken together, these results suggested that KIF4A overexpression was closely related to multiple malignancy-associated clinical characteristics of glioma.

### KIF4A knockdown exhibited therapeutic effects on glioma

**Fig. 4:**
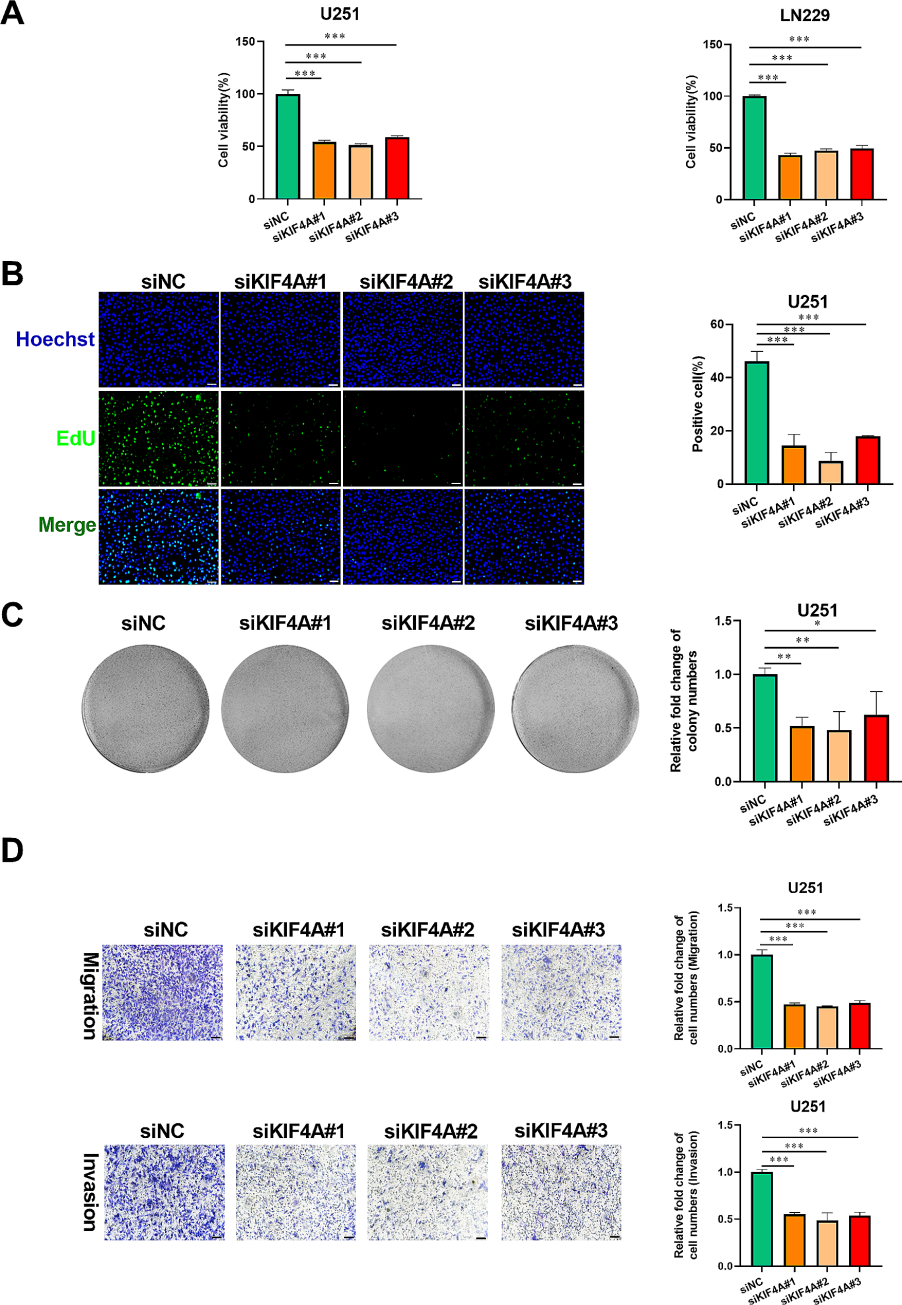
Targeting KIF4A exhibited an antiglioma effect. (**A**) The viability of U251 and LN229 cells transfected with siKIF4A for 48 h was evaluated by an MTT assay. (**B**) The proliferation of U251 cells transfected with siKIF4A for 48 h was evaluated by an EdU incorporation assay. Scale bar: 100 μm. (**C**) The proliferation ability of U251 cells transfected with siKIF4A were evaluated by a colony formation assay. (**D**) The migration and invasion abilities of U251 cells treated with siKIF4A for 48 h were measured by Transwell assays. The bars indicate the means ± SDs; *P < 0.05, **P < 0.01, and ***P < 0.001

Because KIF4A overexpression is associated with poor prognosis in glioma patients, we aimed to demonstrate the effect of KIF4A knockdown on glioma treatment. Subsequently, KIF4A was knocked down in glioma cells (Supplementary Fig. 1A-B). MTT assays were performed to determine the effect of siKIF4A on glioma cell viability, and siKIF4A transfection significantly decreased the viability of U251 and LN229 glioma cells (Fig. 4A). Subsequently, the EdU assay results revealed that the intensity of green fluorescence was markedly decreased by siKIF4A transfection in glioma cell lines (U251 and LN229), indicating that glioma cell proliferation was decreased by siKIF4A (Fig. 4B and Supplementary Fig. 1C). A colony formation assay was subsequently performed to further determine the inhibitory effect of siKIF4A on glioma cell proliferation, and we found that the number of colonies formed was reduced by KIF4A inhibition in glioma cells (Fig. 4C and Supplementary Fig. 1D). In addition, the effects of siKIF4A on glioma cell migration and invasion were quantified by Transwell assays, and as shown in Fig. 4D and Supplementary Fig. 1E, siKIF4A treatment significantly suppressed glioma cell migration and invasion.These observations revealed that KIF4A knockdown exerted an antiglioma effect.

###  WZ-3146 inhibited glioma progression by decreasing KIF4A expression

**Fig. 5:**
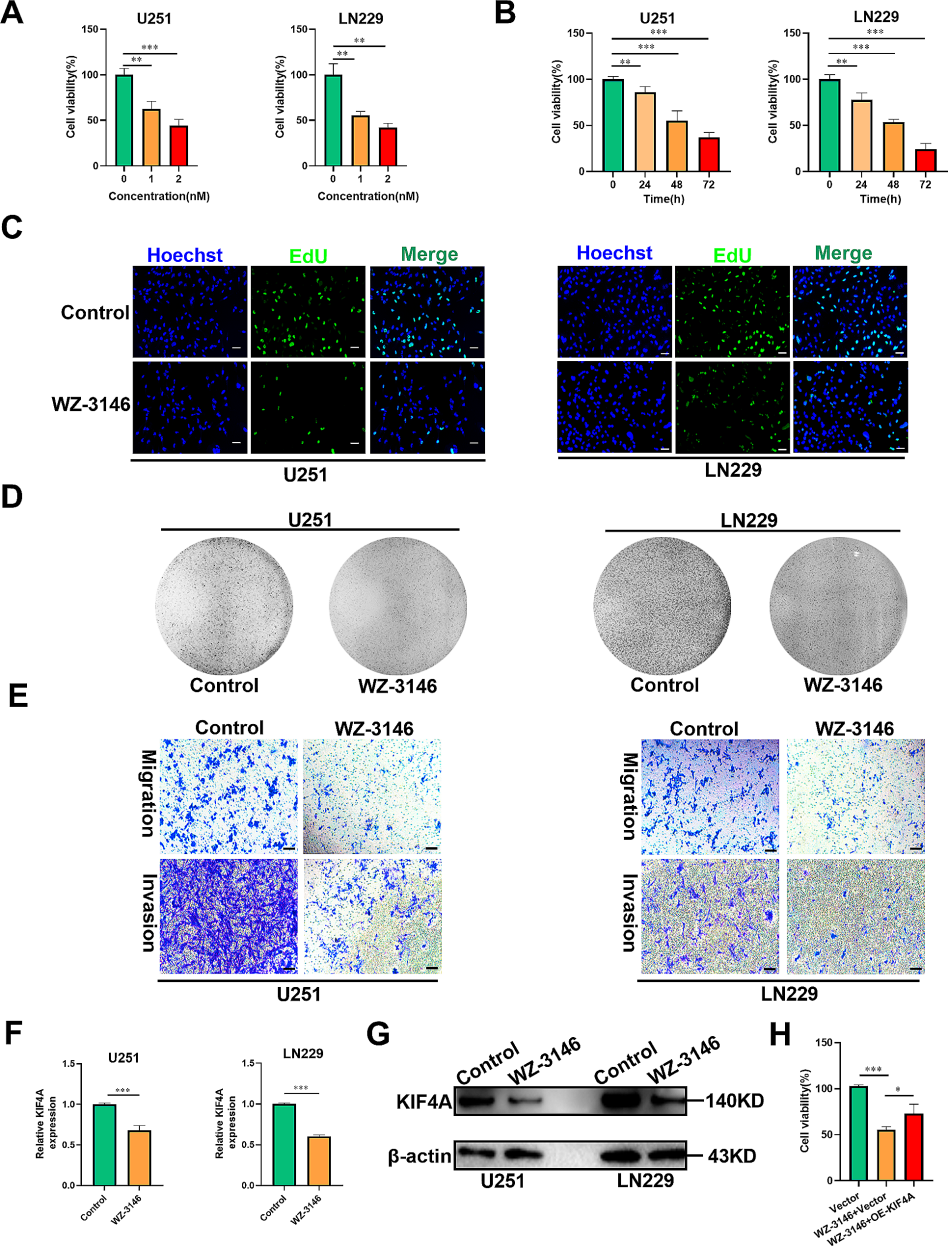
WZ-3146 exerted an inhibitory effect on glioma. (**A**) The viability of U251 and LN229 cells treated with different concentrations of WZ-3146 for 48 h was evaluated by an MTT assay. (**B**) The viability of U251 and LN229 cells treated with WZ-3146 (1nM) for 0, 24, 48 and 72 h was evaluated by an MTT assay. (**C**) The proliferation of U251 and LN229 cells treated with WZ-3146 (1nM) for 48 h were evaluated by an EdU assay. Scale bar: 100 μm. (**D**) The proliferation ability of U251 and LN229 cells treated with WZ-3146 (1nM) were evaluated by a colony formation assay. (**E**) The migration and invasion abilities of U251 and LN229 cells treated with WZ-3146 (1nM) for 48 h were measured by Transwell assays. (**F**) The relative KIF4A expression levels in U251 and LN229 cells treated with WZ-3146 (1nM) for 48 h were measured by qRT‒PCR. (**G**) The relative KIF4A protein levels in U251 and LN229 cells treated with WZ-3146 (1nM) for 48 h were measured by Western blotting. (**H**) The viability of U251 cell line with s overexpressing KIF4A treated by WZ-3146 (1nM) for 48 h was evaluated by an MTT assay. The bars indicate the means ± SDs; *P < 0.05, **P < 0.01, and ***P < 0.001

Given that KIF4A inhibition has an inhibitory effect on glioma, the CMap database (https://clue.io/connectopedia/) was used to identify small molecule inhibitors of KIF4A. First, genes coexpressed with KIF4A in glioma patients in the TCGA database were screened by Pearson correlation analysis. Then, 20 genes positively or negatively correlated with KIF4A expression were uploaded to the CMap database (Supplementary Fig. 2A). Ultimately, WZ-3146 was identified as a small molecule inhibitor that can inhibit KIF4A expression. Moreover, the PubChem database was used to obtain the 2D and 3D structures of WZ-3146 (Supplementary Fig. 2B). To further determine the effect of WZ-3146 on glioma, an MTT assay was performed, and the results demonstrated that WZ-3146 had a dose- and time-dependent lethal effect on glioma cell lines (U251 and LN229), however, WZ-3146 (1nM) had no effect on HUVEC cell activity (Fig. 5A-B, Supplementary Fig. 2C). And the western blot results confirmed that the KIF4A expression in gliomas also showed a dose- and time-dependent on WZ-3146 treatment (Supplementary Fig. 2D). EdU assays revealed that WZ-3146 treatment strongly reduced the intensity of green fluorescence in glioma cell lines (U251 and LN229), suggesting that WZ-3146 can suppress glioma cell proliferation (Fig. 5C and Supplementary Fig. 2E). In addition, consistent with the results of the MTT and EdU assays, the colony formation assay showed that WZ-3146 decreased the colony formation ability of U251 and LN229 cells (Fig. 5D and Supplementary Fig. 2F). In addition, the effects of WZ-3146 on glioma cell migration and invasion were quantified by Transwell assays, and as shown in Fig. 5E and Supplementary Fig. 2G, WZ-3146 treatment significantly suppressed glioma cell migration and invasion. These results indicated that WZ-3146 treatment had antiglioma effects. We then explored whether the inhibitory effect of WZ-3146 on glioma was mediated through the inhibition of KIF4A expression. After WZ-3146 treatment of glioma cell lines (U251 and LN229), KIF4A mRNA and protein expression in these cells was measured by qRT‒PCR and Western blot analyses, and the results demonstrated that KIFA4 expression was strongly decreased by WZ-3146 treatment in glioma cell lines (U251 and LN229) (Fig. 5F-G). Then, KIF4A was over-expressed in glioma cells (Supplementary Fig. 2H). Next, MTT assays suggested that KIF4A overexpression reversed the inhibitory effect of WZ-3146 on proliferation in U251 glioma cell line (Fig. 5H), moreover, the western blot results confirmed that the KIF4A over-expression could reverse the effect of WZ-3146 on KIF4A protein expression in glioma (Supplementary Fig. 2I). According to the above evidences, we hypothesized that WZ-3146 could suppress glioma progression by inhibiting KIF4A expression.

###  WZ-3146 treatment induced glioma cell apoptosis

**Fig. 6:**
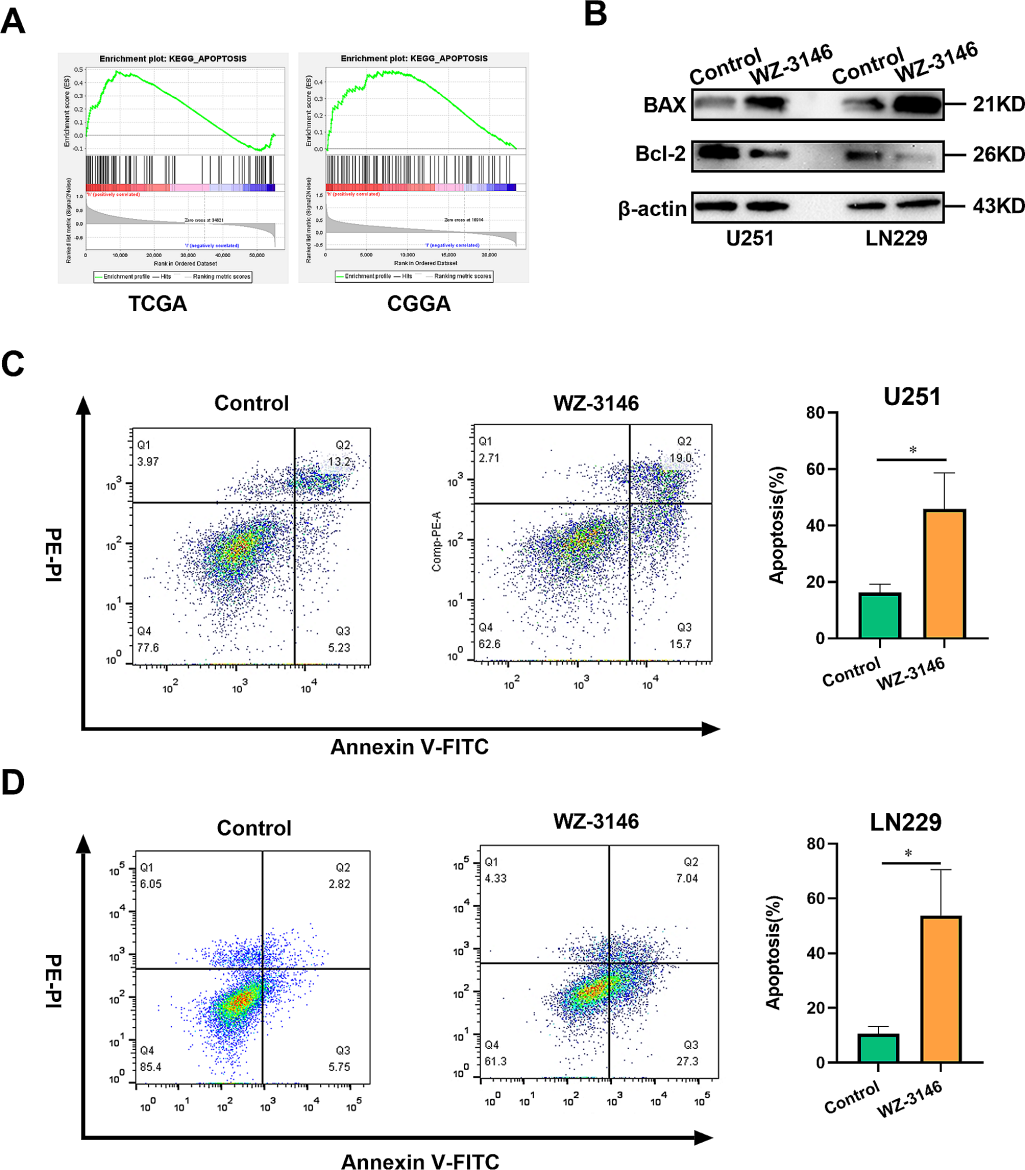
WZ-3146 treatment induced glioma apoptosis. (**A**) GSEA based on the TCGA and CGGA databases indicated that the expression of KIF4A in gliomas is related to cellular apoptosis. (**B**) The relative expression levels of the Bcl-2 and BAX proteins in U251 and LN229 cells were measured by Western blotting after treatment with WZ-3146 (1nM) for 48 h. (**C**-**D**) Apoptosis in U251 and LN229 cells after treatment with WZ-3146 (1nM) for 48 h was detected by flow cytometry. The bars indicate the means ± SDs; *P < 0.05, **P < 0.01, and ***P < 0.001

Considering that KIF4A expression is related to glioma prognosis, gene set enrichment analysis (GSEA) was used to identify the pathways possibly affected by KIF4A. The apoptosis signaling pathway was positively correlated with KIF4A expression in glioma (Fig. 6A). Next, the western blot was used to detced the effect of siKIF4Aon glioma apoptosis, the observations found that the protein level of BAX (a pro-apoptotic factor) was increased and that the protein level of Bcl-2 (an anti-apoptotic factor) was decreased by siKIF4A treatment in glioma cell lines (U251 and LN229) (Supplementary Fig. 2J). Above, we demonstrated that WZ-3146 could inhibit glioma progression via KIF4A inhibition. Therefore, the effect of WZ-3146 on glioma cell apoptosis was explored. The western blot results showed that the protein level of BAX was up-regulated and that the protein level of Bcl-2 (an anti-apoptotic factor) was reduced by WZ-3146 treatment in glioma cell lines (U251 and LN229) (Fig. 6B). More importantly, flow cytometric analysis confirmed that compared with the control treatment, WZ-3146 treatment increased the apoptosis rate of glioma cells (U251 and LN229) (Fig. 6C-D). These results confirmed that WZ-3146 could induce apoptosis. Taken together, these observations reveal that WZ-3146 is highly likely to induce glioma cell apoptosis via KIF4A inhibition.

## Discussion

In the present study, we demonstrated that KIF4A was abnormally overexpressed in glioma and that the overexpression of KIF4A was closely associated with poor prognosis in glioma patients. Moreover, targeting KIF4A could significantly suppress the progression of glioma. Subsequent studies revealed that WZ-3146 can act as a small molecule inhibitor of KIF4A to inhibit glioma cell growth and induce glioma cell apoptosis, ultimately demonstrating a good inhibitory effect on glioma progression. These results supported the idea that WZ-3146 can inhibit the progression of glioma by targeting KIF4A, which, with further research, is anticipated to provide a new adjuvant chemotherapy strategy for glioma.

KIF4A belongs to the kinesin family 4 (KIF4) superfamily; it is localized to condensed and isolated chromosomes and is essential for regulating cell mitosis and meiosis [22]. When KIF4A is absent, the cell cycle checkpoint is activated, leading to inhibition of the attachment of spindles to centromeres, which results in highly condensed chromosomes and, ultimately, abnormal chromosome segregation during cell division [23]. More interestingly, aberrant expression of KIF4A has been found in various tumors; KIF4A is overexpressed in most tumors, but there are also a few tumors with low KIF4A expression [24]. For example, FOXM1 can promote the progression of hepatocellular carcinoma by upregulating KIF4A expression [25]. In breast cancer, adriamycin can induce apoptosis in tumor cells by downregulating the expression of KIF4A [26]. In addition, KIF4A overexpression can promote endometrial cancer progression by inhibiting TPX2 protein degradation [27], while KIF4A expression is reduced in gastric cancer, multiple myeloma, and acute myeloid leukemia [28]. Collectively, these evidences indicate that KIF4A has different functions in different tumors; however, the association of KIF4A expression with glioma has not been comprehensively revealed. Recent research demonstrated that KIF4A knock-down can suppress the glioma stem cells marker expression including CD133, ALDH1A1, and ALDH1A3, meanwhile, KIF4A over-expression can promote the epithelial-to-mesenchymal transition (EMT) of glioma [29]. Here, we confirmed that KIF4A expression is significantly elevated in glioma and negatively correlated with the prognosis of glioma patients by analyses of the TCGA, CGGA, and GEO (GSE4290, GSE50161) databases. Subsequently, univariate and multivariate Cox regression analysis and ROC curve analysis demonstrated that the expression level of KIF4A in glioma can serve as an independent prognostic risk factor for glioma. In addition, further analysis confirmed that the expression level of KIF4A is closely related various clinical characteristics of glioma patients, including the IDH mutation status, 1p19q deletion status, and histological grade. Taken together, these results suggest that overexpression of KIF4A is significantly correlated with poor prognosis in glioma patients. In addition, these results provide preliminary confirmation that targeting KIF4A may constitute a therapeutic approach for glioma.

Considering the different expression patterns of KIF4A in tumors, interfering with KIF4A expression may be a novel strategy for tumor treatment. KIF4A knockdown can reduce esophageal squamous cell carcinoma cell proliferation, migration, and viability [30]. KIF4A knockdown suppresses tumor progression and promotes chemosensitivity in lung cancer [31]. Given that KIF4A overexpression is related to glioma prognosis, we further evaluated the feasibility of targeting KIF4A for glioma treatment. The results confirmed that KIF4A knockdown significantly reduced glioma cell viability and proliferation. These observations indicate that targeting KIF4A can inhibit the malignant progression of glioma. Because KIF4A may be a good target for glioma treatment, we utilized the CMap online database to predict inhibitors of KIF4A, and WZ-3146 was ultimately identified as a potential small molecule inhibitor of KIF4A. Although the effect of WZ-3146 on glioma has not been reported, WZ-3146, as an EGFR inhibitor, can significantly inhibit the growth of pancreatic cancer [32]. Therefore, we speculated that WZ-3146 can inhibit glioma progression by downregulating KIF4A. Our subsequent results demonstrated that WZ-3146 inhibit KIF4A expression in glioma and decreased glioma cell viability and proliferation. Moreover, KIF4A overexpression reversed the inhibitory effect of WZ-3146 on glioma cell activity, suggesting that WZ-3146 can inhibit glioma progression by inhibiting KIF4A expression. Moreover, the ic50 concentration of WZ-3146 (1nM) on glioma was used to detect the effect of WZ-3146 on HUVEC cells, the MTT results indicated that the HUVEC cell activity could not be effected by WZ-3146 at 1nM relative to control group, suggesting WZ-3146 had a specific inhibitory effect on glioma. To further explore the possible pathways affected by WZ-3146 in glioma, we analyzed the effects of KIF4A on glioma signaling pathways by GSEA and found that KIF4A significantly affects the apoptosis signaling pathway in glioma, consistent with the finding that KIF4A knockdown can induce prostate and ovarian cancer cell apoptosis [33, 34]. Therefore, we further examined the effect of WZ-3146 on glioma cell apoptosis, and the results confirmed that WZ-3146 can significantly induce glioma cell apoptosis, which further confirmed that WZ-3146 can limit glioma progression by targeting KIF4A. Overall, we believe that through future in-depth research, WZ-3146 may be used for adjuvant chemotherapy for glioma.Meanwhile, we have only demonstrated that WZ-3146 could suppress glioma progression by inhibiting KIF4A expression in vitro. In future study, we will further explore the important role of KIF4A expression in glioma progression, especially the funciton of KIF4A in the construction of glioma immunosuppressive microenvironment, through combining animal tumor models and gene mutation technique, which is one of the research directions in our research.

However, the mechanism by which WZ-3146 acts as an EGFR inhibitor to inhibit KIF4A expression in glioma remains unclear. An increasing number of studies have reported that EGFR may regulate KIF4A expression, activating downstream signal transduction pathways such as the STAT3, ERK, and mTOR pathways [35]. This indirect evidence preliminarily proves that WZ-3146 may suppress EGFR expression, ultimately inhibiting KIF4A expression, in glioma. However, the specific molecular mechanism by which WZ-3146 inhibits KIF4A expression in glioma is not clear and is the focus of our next study.

In conclusion, our findings indicated that KIF4A overexpression in glioma is associated with poor prognosis and that targeting KIF4A can inhibit glioma progression. WZ-3146, a small molecule inhibitor of KIF4A, can inhibit glioma growth and induce glioma cell apoptosis, thus exerting antiglioma effects. Therefore, WZ-3146 is worthy of further study as an active antiglioma molecule. We speculate that WZ-3146 may be a novel drug targeting KIF4A that could be use in combination with other targeted drugs, which may be an effective chemotherapeutic strategy for glioma.

### Electronic supplementary material

Below is the link to the electronic supplementary material.

Supplementary Material 1

Supplementary Material 2

Supplementary Material 3

## Data Availability

No datasets were generated or analysed during the current study.
