# Supplementary material for: WZ-3146 acts as a novel small molecule inhibitor of KIF4A to inhibit glioma progression by inducing apoptosis
Source: Cancer Cell Int. 2024 Jun 27;24:221. doi: 10.1186/s12935-024-03409-y (PMC11209999; doi:10.1186/s12935-024-03409-y)
Supplement: Supplementary file 1 — Supplementary Material 1 [file 12935_2024_3409_MOESM1_ESM.docx]

**Supplementary Figure 1**

(A-B) Gene knockdown efficiency of the KIF4A siRNA in U251 glioma cell line was measured by qRT-PCR and Western bloting. (C) The proliferation of LN229 cells transfected with siKIF4A for 48 h was evaluated by an EdU incorporation assay. Scale bar: 100 μm. (D) The proliferation ability of LN229 cells transfected with siKIF4A were evaluated by a colony formation assay. (E) The migration and invasion abilities of LN229 cells treated with siKIF4A for 48h were measured by Transwell assays. The bars indicate the means±SDs; *P<0.05, **P<0.01, and ***P<0.001.

**Supplementary Figure 2**

1. List of the top 20 genes with positive and negative co-expression relationships with KIF4A in the TCGA database. (B) The 3D structure, 2D structure, molecular formula and PubChem CID of WZ-3146 was predicted by CMap analysis. (C) The viability of HUVEC cells treated with WZ-3146 (1nM) for 48h was evaluated by an MTT assay. (D) The protein levels of KIF4A expression in U251 and LN229 cells treated with different concentrations of WZ-3146 for 48h was evaluated by Western blot, and the protein levels of KIF4A expression in U251 and LN229 cells treated with WZ-3146 (1nM) for 0, 24, 48 and 72h was evaluated by Western blot. (E) The Statistical graph of the EDU assay in Figure 5C. (F) The Statistical graph of the colony formation assay in Figure 5D. (G) The Statistical graph of the transwell assay in Figure 5E. (H) Gene over-expressed efficiency of the KIF4A overexpressed plasmid in U251 glioma cell line was measured by qRT-PCR and Western bloting. (I) The protein levels of KIF4A expression in U251 cells was evaluated by Western blot, after treatment with WZ-3146 , followed by treatment with the KIF4A overexpressed plasmid for 48h. (J) The relative expression levels of the Bcl-2 and BAX proteins in U251 and LN229 cells were measured by Western blotting after treatment with siKIF4A for 48 h. The bars indicate the mean±SD;*p<0.05,**p<0.01,and**=p<0.001.
