# Supplementary figures and images for: WZ-3146 acts as a novel small molecule inhibitor of KIF4A to inhibit glioma progression by inducing apoptosis
Source: Cancer Cell Int. 2024 Jun 27;24:221. doi: 10.1186/s12935-024-03409-y (PMC11209999; doi:10.1186/s12935-024-03409-y)

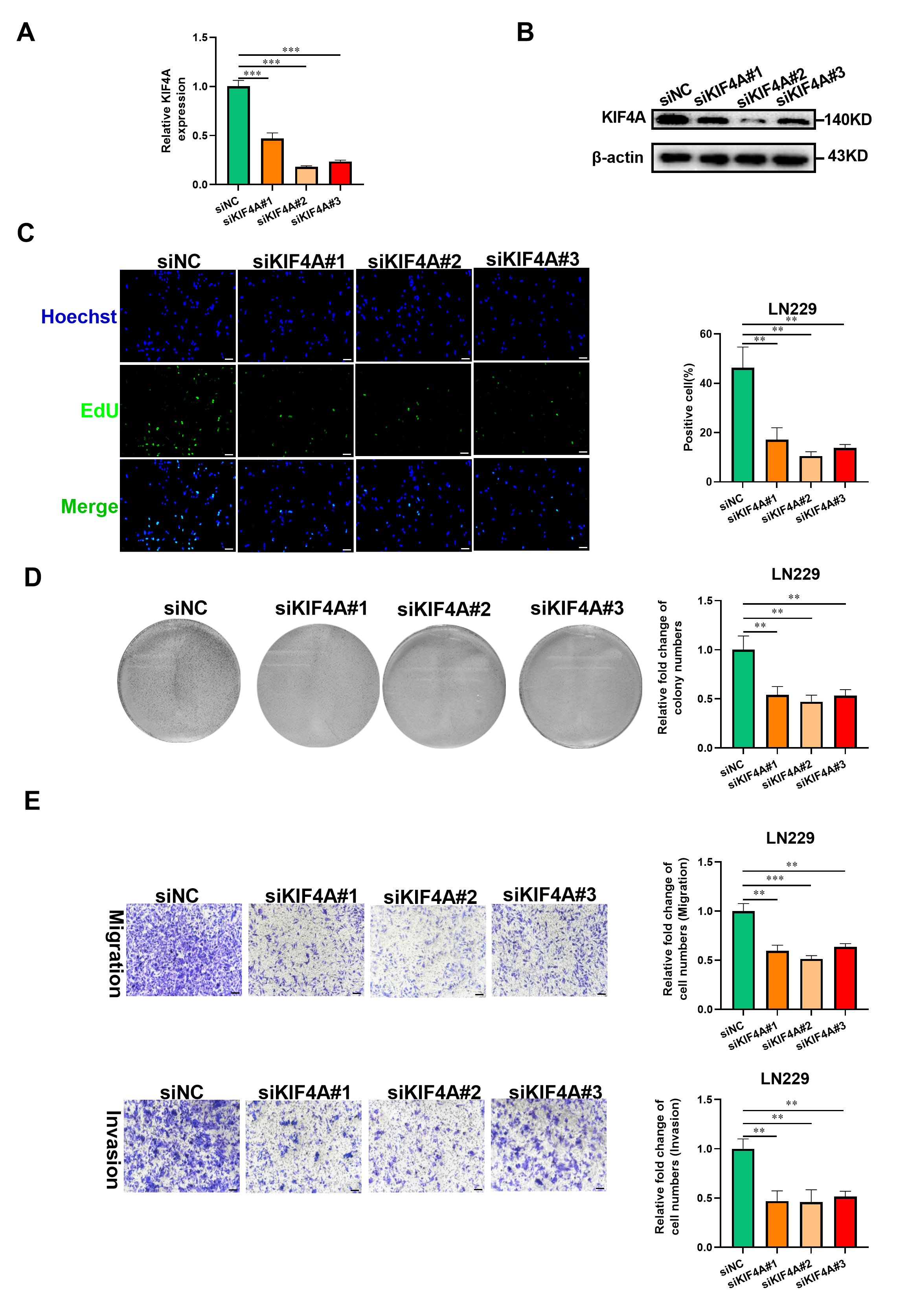

Supplement: Supplementary file 2 — Supplementary Material 2 [file 12935_2024_3409_MOESM2_ESM.png]

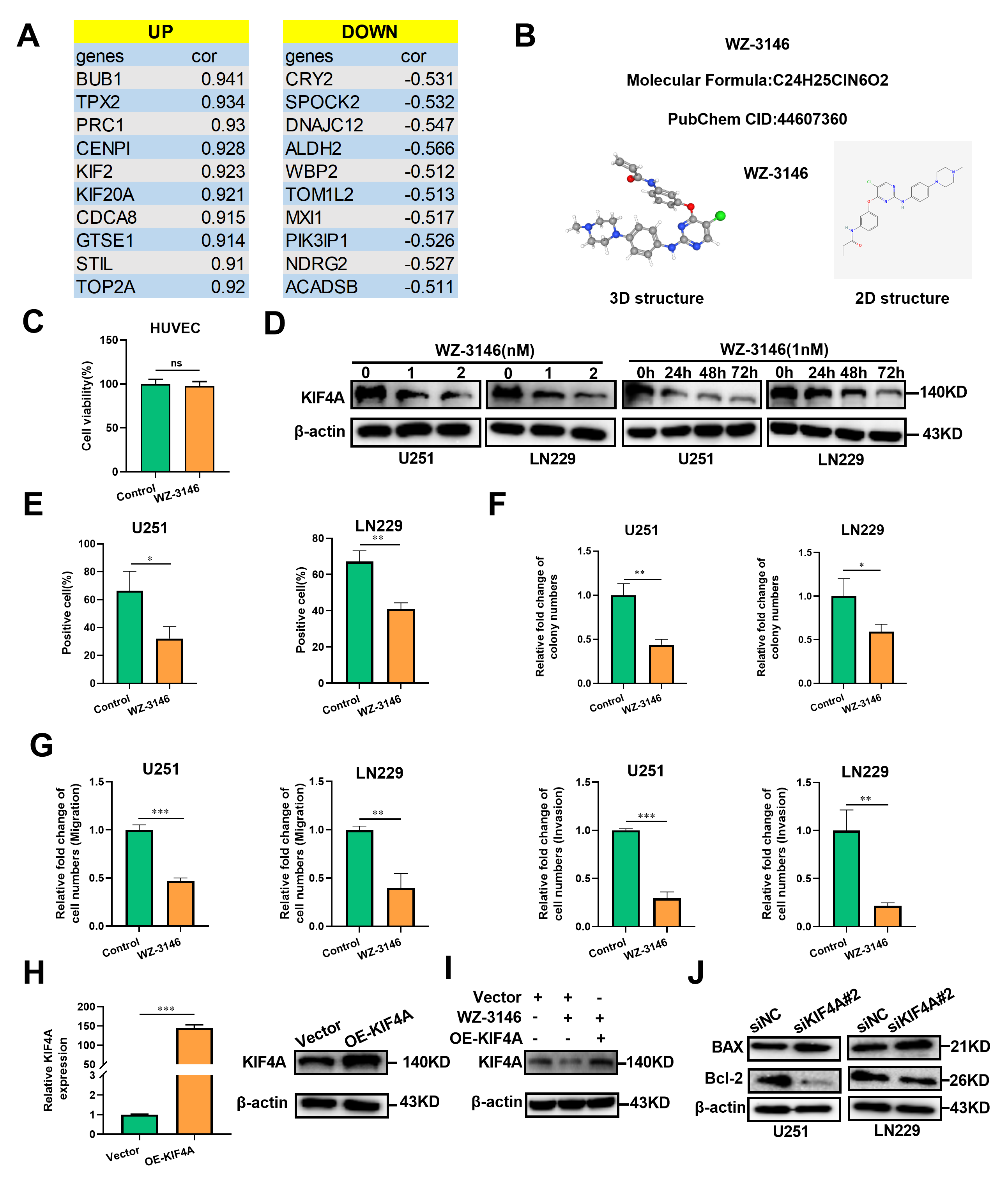

Supplement: Supplementary file 3 — Supplementary Material 3 [file 12935_2024_3409_MOESM3_ESM.png]
